# Increasing the Pentose Phosphate Pathway Flux to Improve Plasmid DNA Production in Engineered *E. coli*

**DOI:** 10.3390/microorganisms12010150

**Published:** 2024-01-12

**Authors:** Mitzi de la Cruz, Flavio Kunert, Hilal Taymaz-Nikerel, Juan-Carlos Sigala, Guillermo Gosset, Jochen Büchs, Alvaro R. Lara

**Affiliations:** 1 Departamento de Procesos y Tecnología, Universidad Autónoma Metropolitana, Mexico City 05348, Mexico; 2 Chair of Biochemical Engineering (AVT.BioVT), RWTH Aachen University, 52074 Aachen, Germany; 3 Department of Genetics and Bioengineering, Istanbul Bilgi University, 34060 Istanbul, Turkey; 4 Instituto de Biotecnología, Universidad Nacional Autónoma de México, Cuernavaca 62210, Mexico; 5 Department of Biological and Chemical Engineering, Aarhus University, 8000 Aarhus, Denmark

**Keywords:** plasmid DNA vaccines, pentose phosphate pathway, glucose-glycerol co-utilization

## Abstract

The demand of plasmid DNA (pDNA) as a key element for gene therapy products, as well as mRNA and DNA vaccines, is increasing together with the need for more efficient production processes. An engineered *E. coli* strain lacking the phosphotransferase system and the pyruvate kinase A gene has been shown to produce more pDNA than its parental strain. With the aim of improving pDNA production in the engineered strain, several strategies to increase the flux to the pentose phosphate pathway (PPP) were evaluated. The simultaneous consumption of glucose and glycerol was a simple way to increase the growth rate, pDNA production rate, and supercoiled fraction (SCF). The overexpression of key genes from the PPP also improved pDNA production in glucose, but not in mixtures of glucose and glycerol. Particularly, the gene coding for the glucose 6-phosphate dehydrogenase (G6PDH) strongly improved the SCF, growth rate, and pDNA production rate. A linear relationship between the G6PDH activity and pDNA yield was found. A higher flux through the PPP was confirmed by flux balance analysis, which also estimates relevant differences in fluxes of the tricarboxylic acid cycle. These results are useful for developing further cell engineering strategies to increase pDNA production and quality.

## 1. Introduction

Plasmid DNA (pDNA) is an essential component of DNA vaccine and gene therapy products [1], as well as for the manufacturing of mRNA vaccines [2]. pDNA production has been identified as the bottleneck for successful development and commercialization of genetic medicine products [3]. Therefore, there is a high need to develop strategies to improve pDNA production, as well as advance the understanding of the factors limiting pDNA synthesis. pDNA is normally produced using *Escherichia coli* as host, and the typical challenges associated to *E. coli* cultures, like overflow metabolism (aerobic acetate production) [4,5], remain. Several genetic interventions have been carried out to improve pDNA production and to reduce overflow metabolism in *E. coli*. Because the supercoiled isoform is presumably more efficient for pDNA transfection [1], the Food and Drug Administration of the United States of America has recommended that the supercoiled fraction (SCF) of the pDNA intended for therapeutical applications should be at least 80% [6]. Therefore, genetic interventions have also been performed to increase the yield and SCF of the produced pDNA. Among others, such modifications include deleting the genes *recA* (coding for the recombinase A) and *pykA* (coding for the pyruvate kinase A) to increase the stability of the pDNA supercoiling and increase the carbon flux to the pentose phosphate pathway (PPP) [7,8], respectively. Increasing the flux to the PPP has been addressed due to the synthesis of DNA precursors in this pathway. Therefore, the *zwf* and *rpiA* genes (coding the glucose-6-phosphate dehydrogenase and ribose-5-phosphate isomerase A, respectively) have been overexpressed [7,9,10,11]. The published studies do not present all the information about pDNA yields, productivities, or SCF, and in some cases, the published results can be contradictory [9,10,11]. Moreover, some of these studies used highly specialized *E. coli* strains, optimized media, and thermal induction. Consequently, it remains unclear whether the proposed strategies can be transferred to other strains of interest.

In a particular *E. coli* strain, the phosphotransferase system was substituted by the galactose permease (PTS^−^ GalP^+^), which displays strongly reduced overflow metabolism. This strain was cultured to high cell-densities using up to 100 g L^−1^ glucose in batch mode for pDNA production, with little acetate accumulation [12]. The strain was further engineered to increase pDNA yields and was cultured to high cell-densities in batch mode, reaching nearly 200 mg L^−1^ of an experimental DNA vaccine against mumps [13]. The *pykA* gene was deleted in this strain, which increased its biomass yield from glucose and eliminated the production of acetate [14]. This strain, named VH34, was cultured in chemostats and displayed higher pDNA yields from biomass (Y_pDNA/X_) and specific pDNA production rates (*q_pDNA_*), at dilution rates between 0.16 and 0.23 h^−1^, than its wild type (*E. coli* W3110) [15]. Consequently, strain VH34 is a good candidate to produce pDNA in high cell-density cultures in batch mode or at relatively low dilution rates. Despite these results, a disadvantage of strain VH34 is a slow growth rate compared to its parental strain. This could be overcome by the addition of a carbon source (C-source) additional to glucose, like glycerol. Because strain VH34 lacks catabolite repression, both C-sources can be consumed simultaneously. In this study, we present a systematic study of the effects of glycerol and glucose co-consumption, in combination with *zwf* or *rpiA* overexpression, as strategies to increase the carbon flux to the PPP and improve pDNA production in strain VH34.

## 2. Materials and Methods

### 2.1. Strains and Plasmids

The K-12 derivative W3110 *Escherichia coli* was used as the wild type. Strain VH34 is a Δ*ptsI*, *ptsH*, *crr*::*km*, ΔP*_galP_*::P*_trc_*, *pykA*::*cat* derivative of W3110 [14]. The *recA* gene was further deleted in each strain as described elsewhere [16]. The strain VH34recA^−^ is named here as VH36. The strains were transformed with the plasmids described below. Transformed and untransformed strains were plated in Petri dishes and grown at 37 °C for 14–18 h. One colony per strain was selected and grown in Terrific Broth (TB) medium until reaching an optical density at 600 nm (OD_600_) of approx. 6–8. Then, 0.9 mL of such culture broth was mixed with a sterile solution of glycerol (80% *v*/*v*) and immediately frozen at −80 °C.

The different strains were transformed with the plasmid pUC57mini (1835 bp), which confers ampicillin resistance. The plasmids pUC57mini-zwf (4975 bp) or pUC57mini-rpiA (4124 bp) contain the sequence of the *zwf* or the *rpiA* genes under transcriptional control of the P*_trc_* promoter. The complete sequences were synthesized and cloned in the plasmid pUC57mini by GenScript (Piscataway, NJ, USA).

### 2.2. Precultures Development

A first preculture was performed by transferring 0.05 mL of the cryopreserved cells to 8 mL of TB medium contained in a 250 mL Erlenmeyer flask. The composition of TB was (in g L^−1^): yeast extract (Sigma-Aldrich, St. Louis, MO, USA), 24; tryptone (Sigma-Aldrich, St. Louis, MO, USA), 20; glycerol, 3.2. The cells were grown at 37 °C and 350 rpm in an orbital shaker of 50 mm shaking diameter for 6 h. Next, 0.05 mL was transferred to 8 mL of a mineral medium contained in a 250 mL Erlenmeyer flask and cultured at 30 °C and 350 rpm in an orbital shaker of 50 mm shaking diameter until the mid-exponential growth phase (approx. 14–16 h). For precultures and main cultures of the untransformed strains, the mineral medium composition was (in g L^−1^): (NH_4_)_2_SO_4_, 6.5; NH_4_Cl, 0.5; K_2_HPO_4_, 3.0; Na_2_SO_4_, 2.0; MgSO_4_·7H_2_O, 0.5; thiamine hydrochloride, 0.01; MOPS, 41.85; plus 1 mL of trace elements solution per L of medium. The initial pH was adjusted to 7.4. The trace elements solution composition was (in g L^−1^): ZnSO_4_·7H_2_O, 0.54; CuSO_4_·5H_2_O, 0.48; MnSO_4_·H_2_O, 0.30; CoCl_2_·6H_2_O, 0.54; FeCl_3_·6H_2_O, 41.76; CaCl_2_·2H_2_O, 1.98; Na_2_EDTA·2H_2_O, 33.4. For cultures of plasmid-bearing cells, the mineral medium composition was (in g L^−1^): K_2_HPO_4_, 17; KH_2_PO_4_, 5.3; (NH_4_)SO_4_, 2.5; NaCl, 1.0; MgSO_4_·7H_2_O, 1.0; thiamine hydrochloride, 0.01; ampicillin sodium salt, 0.1; plus 2.5 mL L^−1^ trace elements solution. The trace elements solution composition was (in g L^−1^): ZnCl_2_, 10.5; EDTA, 5.5; CoSO_4_·7H_2_O, 1.5; MnSO_4_·H_2_O, 6.4; CuSO_4_·5H_2_O, 1.1; H_3_BO_3_, 1.5; Na_2_MoO_4_·2H_2_O, 1.0; FeCl_3_·6H_2_O, 51.4; Cit-H·H_2_O, 39.9. The same main C-source to be used in the main culture was added at a final concentration of 10 g L^−1^. Aliquots from the second preculture were washed with 0.9% sterile NaCl solution and used to inoculate the main cultures.

### 2.3. Culture Conditions

The untransformed strains were grown in 250 mL Erlenmeyer flasks containing 30 mL of the mineral medium plus 3 g L^−1^ glucose, glycerol, or a mixture of 1.5 g L^−1^ each, at 37 °C and 350 rpm in an orbital shaker of 50 mm shaking diameter. In parallel and using the same precultures and shaker, the oxygen transfer rates (OTR) were determined online using the Respiration Activity Monitoring System (RAMOS) [17] in independent flasks. The plasmid-bearing strains were cultured in 250 mL baffled shake flasks (4 baffles, 30 mm height, located at the bottom of the flask) filled with 50 mL of medium supplemented with 1.5 g L^−1^ glucose or glycerol, or 1 g L^−1^ of each C-source in mixtures. IPTG was added prior to inoculation for inducing the *rpiA* or *zwf* genes. The initial OD_600_ was 0.3 units. Culture conditions were 37 °C, 250 rpm, and 50 mm shaking diameter. Cell growth was followed by OD_600_ measured in a biophotometer (Eppendorf, Hamburg, Germany).

### 2.4. Quantification of Concentration of Metabolites

Glucose, glycerol, and acetate concentrations were measured via a high-performance liquid chromatography system (Shimadzu Prominence LC-20, Duisburg, Germany) equipped with a precolumn Organic Acid Resin (40 × 8 mm, CS-Chromatographie Service, Langerwehe, Germany), a separating column Organic Acid Resin (250 × 8 mm, CS-Chromatographie Service, Langerwehe, Germany), and a refraction index detector RID-20A (Shimadzu, Duisburg, Germany). The flow rate of the mobile phase (5 mM H_2_SO_4_) was set to 0.8 mL/min with a column temperature of 50 °C. For cultures of strain-bearing plasmids, glucose was measured using a YSI 2950D Biochemistry Analyzer (YSI Inc., Yellow Springs, OH, USA).

### 2.5. Plasmid DNA Analyses

Approximately 2 mg of wet biomass was used for pDNA purification using a QIAprep Miniprep Kit (Qiagen, Hilden, Germany), according to the manufacturer’s recommendations. pDNA was eluted using 80 µL of TE buffer at 70 °C to enhance its recovery. pDNA was quantified by UV spectroscopy in a NanoDrop 2000 spectrophotometer (Thermo Scientific, Walthman, MA, USA). The SCF was determined by electrophoresis of 100 ng of pDNA in 1% agarose gel in TAE buffer for 1 h at 80 V. A sample of linear plasmid, obtained by digesting a sample with the enzyme BamHI (Invitrogen, Carlsbad, CA, USA), was also loaded. Supercoiled pDNA was identified by the bands that migrate faster than the linear plasmid and correspond to the covalently closed circular (ccc) monomer [18]. Image analyses were performed with the Image J software (NIH, Bethesda, MD, USA). Therefore, the supercoiled fraction (SCF) here is referred to as the ccc monomer. Other isoforms were not quantified.

### 2.6. Measurement of the Glucose-6-Phosphate 1-Dehydrogenase (G6PDH) Activity

Approx. 1.5 g of wet biomass were taken from exponentially growing cultures and washed twice at 4 °C with a lysis buffer, consisting of the following components at a concentration of 0.01 mol L^−1^ each: KH_2_PO_4_, Na_2_HPO_4_, mercaptoethanol, and sodium azide, pH = 6.8. After the second wash, biomass was resuspended in 2 mL of lysis buffer and subjected to sonication, using an amplitude of 35% and two pulses (15 s sonication followed by 15 s rest, and then 1 min sonication and 1 min rest) employing a CV33 GENTEQ ultrasonic processor (IN, USA). The resulting solution was centrifuged at 14,000 rpm and 4 °C for 15 min, and the pellet was resuspended in 2 mL of lysis buffer. The total protein content of this solution was measured using the Bradford protocol [19]. The G6PDH activity was measured from 500 µL of the lysed biomass using the Glucose-6-Phosphate Dehydrogenase Assay Kit MK015 from Sigma-Aldrich (St. Louis, MO, USA), following the instructions of the manufacturer. The principle of the kit is to detect the oxidation of NADH by the glucose-6-phosphate by a colorimetric (450 nm) assay.

### 2.7. Calculations

Specific yields and rates were calculated during the exponential growth phase. To calculate the specific consumption rates in cultures with simultaneous consumption of glucose and glycerol, the change of each carbon source concentration over time was described by a polynomial fit. Next, the first derivative was calculated and divided by the biomass concentration for every data point and the average specific uptake rate was calculated.

### 2.8. Flux Balance Analysis

Flux balance analysis (FBA) was carried out using an *E. coli* genome scale model [20] in a COBRA toolbox [21], maximizing biomass formation. For constraints of FBA, measured substrate uptake rates of glucose and glycerol and production rate of acetate were implemented for each strain. In the presence of glycerol, ubiquinone-8-dependent glycerol-3-phosphate dehydrogenase (G3PD5) was used in the model [22]. For strain VH34, the flux of D-glucose transport via PEP:Pyr PTS (GLCptspp) was set to zero in all cases. Additionally, the flux of glycerol dehydrogenase (GLYCDx) was set to zero, since it was reported that this reaction is utilized under anaerobic consumption of glycerol, but not aerobically [23].

## 3. Results

### 3.1. Cultures of Untransformed Strains W3110 and VH34 in Mixtures of Glucose and Glycerol

**Figure 1:**
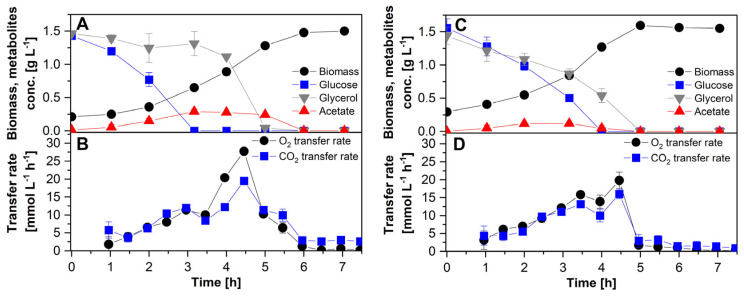
(**A**,**B**): Diauxic growth of the strain W3110 in a mixture of glucose and glycerol. (**C**,**D**): Growth of strain VH34 simultaneously consuming glucose and glycerol.

The untransformed strains W3110 and VH34 were cultured in C-source mixtures containing 1.5 g L^−1^ glucose + 1.5 g L^−1^ glycerol. The culture profiles are shown in Figure 1. The wild-type strain W3110 consumed glucose with the concomitant production of acetate. Only when glucose was exhausted, after approx. 3 h of culture, was glycerol consumed, accompanied by reassimilation of acetate (Figure 1A). This diauxic shift can also be observed in the rates of CO_2_ and O_2_ transfer (Figure 1B). Glycerol and glucose were consumed simultaneously in cultures of the engineered strain VH34, while acetate was produced (Figure 1C), although at lower levels compared to its parental strain. Glucose was consumed approx. 1 h earlier than glycerol. The shift from the simultaneous consumption of glucose and glycerol, to consumption of glycerol as the only C-source, was also reflected in the profiles of CO_2_ and O_2_ transfer (Figure 1D).

The strains were also cultured using 1.5 g L^−1^ of each C-source independently. The extracellular rates were calculated during the exponential growth phase and are shown in Table 1. The growth of strain VH34 in glucose was slower than that of its parental strain, which is a result of the lower glucose uptake rate (*q_glucose_*), while acetate production by overflow metabolism was totally prevented in strain VH34. These metabolic states agree with the respiratory quotient (RQ) values measured. The specific growth rate in glycerol was lower for both strains compared to growth in glucose. The specific acetate production rate of strain W3110 growing in glycerol was less than one-half of the corresponding value in glucose, while strain VH34 did not produce acetate at all. The RQ values were similar for both strains.

When growing in a mixture of glucose and glycerol, strain W3110 consumed glucose at the same rate as in cultures with glucose as the only C-source, but acetate production was faster and RQ was higher. This implies that the presence of both carbon sources caused changes to the metabolism to generate more CO_2_ per mol of oxygen and thus reduced the biomass yield from glucose. Glucose and glycerol were consumed simultaneously by strain VH34 at rates lower than in cultures with a single C-source. However, the total C-source consumption was similar to the glycerol uptake rate as the only C-source. This agrees with the study of Okano and coworkers [24], in which it was proposed that in C-source mixtures simultaneously consumed by *E. coli*, the glycerol uptake rate responds to the total carbon uptake flux, which remains at a level corresponding to that of growth on glycerol alone. The simultaneous consumption of glucose and glycerol increased the specific growth rate of VH34 compared to cultures using only one C-source, while the specific acetate production rate (*q_acetate_*) was very low. This contrasts with the results from Yao and coworkers [25], who reported that the *q_acetate_* was higher for a Δ*pts* GglpK *E. coli* strain growing in glucose-glycerol mixtures than for its parental strain. Therefore, the simultaneous consumption of glucose and glycerol has a positive impact on the growth of strain VH34, with only minor effects on overflow metabolism.

### 3.2. Effect of recA Inactivation on pDNA Production in Strain VH34

It has been shown that the deletion of the gene coding for the DNA recombination/repair protein RecA increases the SCF [16,26]. Therefore, the effect of deleting *recA* in strain VH34 was tested and compared to strain W3110recA^−^, which was taken as a reference strain for pDNA production with a natural glucose transport system. It has already been demonstrated that *recA* deletion in W3110 lowers the Y_pDNA/X_ but increases the SCF [26]. The strain VH34recA^−^ is named here as VH36. The strains were transformed with the high copy-number plasmid pUC57mini and cultured in a mineral medium with glucose as the only C-source. The main parameters are reported in Table 2. Strain W3110recA^−^ displayed high specific growth, glucose uptake, and acetate production rates. The SCF of the produced pDNA was close to 80%, which is the minimum recommended by the Food and Drug Administration for therapeutic use [6]. The strain VH34 grew much slower than W3110recA^−^ and produced no acetate. The Y_pDNA/X_ in cultures of strain VH34 was 14% higher than in cultures of W3110recA^−^, however, the SCF was extremely low. Moreover, due to the slow growth rate, the specific rate of pDNA production (*q_pDNA_*) was only one-third of the attained in cultures of strain W3110recA^−^.

The deletion of *recA* in VH34 had no effect on the growth rate and a negligible effect on acetate production. The Y_pDNA/X_ was 28% lower in cultures of VH36 than in cultures of W3110recA^−^. However, the SCF increased strongly compared to VH34, although it was low compared to the regulatory guidelines. Therefore, the next experiments were aimed at improving the production of pDNA in strain VH36 considering not only the yield and rates, but also the quality of the pDNA produced.

### 3.3. Strategies to Improve pDNA Production in Strain VH36

**Figure 2:**
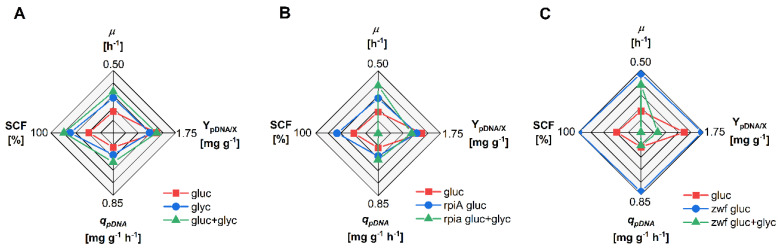
Comparison of the main parameters for pDNA production in cultures of strain VH36 in glucose (gluc), glycerol (glyc), or a mixture of glucose and glycerol (gluc + glyc). The data correspond to cultures of strains (**A**): VH36; (**B**): VH36rpiA; (**C**): VH36zwf. *µ*: specific growth rate; Y_pDNA/X_: pDNA yield from biomass; *q_pDNA_*: specific pDNA production rate; SCF: pDNA supercoiled fraction. The performance of VH36 (red squares) is shown in all panels for easier comparison.

Three different strategies were evaluated to improve pDNA production in strain VH36. With the aim of increasing the carbon flux to the PPP, strain VH36 was cultured in a mixture of glucose + glycerol. Additionally, the genes *zwf* or *rpiA* were overexpressed. Cultures were carried out in a mineral media as described before. To induce the expression of *zwf* and *rpiA*, IPTG was added to the medium prior to inoculation, to a final concentration of 0.1 mM. Figure 2 shows the values of the main parameters calculated during the exponential growth phase of the different cultures. Figure 2A shows the effect of independent C-sources or their mixture on the main parameters. The value of Y_pDNA/X_ was slightly lower, but *µ* was greater in glycerol than in glucose, which consequently resulted in a higher *q_pDNA_* value in glycerol than in glucose. Interestingly, the SCF increased from 39 ± 4% in glucose to 69 ± 3% in glycerol. When glucose and glycerol were simultaneously consumed, the value of *µ* was 0.33 ± 0.01 h^−1^, which was higher than using glucose or glycerol separately. The value of Y_pDNA/X_ was similar in C-source mixtures, compared to cultures in glucose, which increased the *q_pDNA_* up to 0.40 ± 0.02 mg g^−1^ h^−1^. Moreover, the co-consumption of glucose and glycerol raised the SCF up to 79 ± 1%. These results show that the simultaneous consumption of glucose and glycerol is an attractive option to improve pDNA production in strain VH36. Therefore, this strategy was combined with the overexpression of key genes of the PPP.

The effects of overexpressing the *rpiA* gene in cultures of VH36 (denoted as VH36rpiA) using glucose or a mixture of glucose and glycerol are shown in Figure 2B. The results of cultures of VH36 in glucose (no *rpiA* overexpression) are included for an easier comparison. Overexpression of *rpiA* in cultures with glucose as the only C-source resulted in a reduced Y_pDNA/X_, but higher *µ*, compared to cultures of VH36 in glucose. However, *q_pDNA_* reached a value of 0.31 ± 0.02 g g^−1^ h^−1^ and the SCF was 66 ± 1%. These values, although higher than those corresponding to cultures of VH36 in glucose, were lower than those obtained in cultures of VH36 in C-source mixtures (Figure 2A). The overexpression of *rpiA* during the co-consumption of glycerol and glucose strongly increased *µ*, which reached 0.38 ± 0.00 h^−1^. However, Y_pDNA/X_ decreased to 0.94 ± 0.20 mg g^−1^, which did not improve the value of *q_pDNA_*, compared to cultures of strain VH36 overexpressing the gene *rpiA* in glucose as the only C-source. The produced pDNA was predominantly found as multimers, which reduced the SCF to nearly zero. Overall, overexpressing *rpiA* in VH36 is beneficial for SCF and *µ* when glucose is the only C-source, while Y_pDNA/X_ and *q_pDNA_* are not increased. If glucose and glycerol are used together, the main benefit is faster biomass formation.

Overexpression of the gene *zwf* in VH36 (denoted as VH36zwf) strongly improved pDNA production using glucose as the only C-source, in comparison to cultures of VH36 without *zwf* overexpression (Figure 2C). Namely, *µ*, Y_pDNA/X_, and *q_pDNA_* reached values of 0.47 ± 0.01 h^−1^, 1.71 ± 0.17 mg g^−1^, and 0.81 ± 0.06 mg g^−1^ h^−1^, respectively. This represents a four-fold increase of pDNA-specific productivity compared to cultures of VH36 in glucose without *zwf* overexpression. Moreover, the overexpression of *zwf* using glucose as the only C-source increased the SCF to 100% (Figure 2C), which represents a relevant improvement when compared to the strain W3110recA^−^ (Table 2). Notwithstanding these positive results in glucose, when glycerol and glucose were co-consumed, the overexpression of *zwf* was detrimental for pDNA production, compared to cultures of VH36 in glucose. In this case, the only positive effect was an increase of *µ* up to 0.38 ± 0.01 h^−1^. As in the case of *rpiA* overexpression, no supercoiled pDNA was detected when glucose and glycerol were co-consumed.

**Figure 3:**
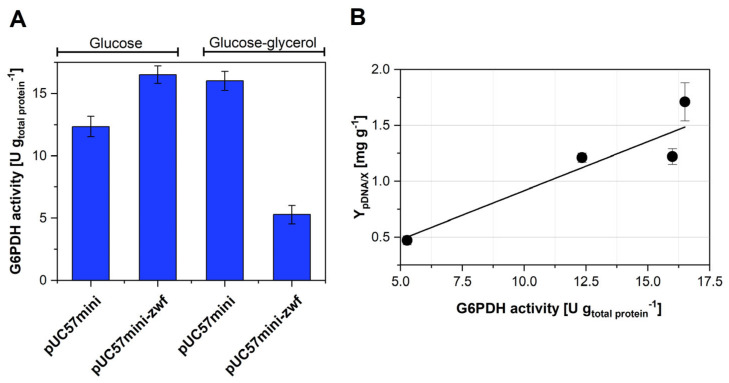
G6PDH activity and its effect on pDNA yields. (**A**): Impact of mixed C-sources and *zwf* overexpression on G6PDH activity in strain VH36. The names of the plasmids used are indicated in the horizontal axis. (**B**): Relationship between the G6PDH activity and the plasmid yield from biomass (Y_pDNA/X_).

The overexpression of the *zwf* gene using glucose as the only C-source was the best option so far to improve pDNA production in strain VH36. To gain more knowledge in this regard, the GPPDH activity was measured in cultures of strains VH36 and VH36zwf using either glucose or mixtures of glucose and glycerol. As shown in Figure 3A, the G6PDH activity was 34% higher in strain VH36zwf than in strain VH36 when glucose was the only C-source. The simultaneous consumption of glucose and glycerol resulted in 30% higher G6PDH activity in strain VH36. However, when both C-sources were simultaneously consumed and *zwf* overexpressed, the G6PDH was 60% lower than in cultures of strain VH36 using glucose as the only C-source. Figure 3B shows the Y_pDNA/X_ values plotted against the corresponding G6PDH activity. There seems to exist a linear relationship between the G6PDH activity and the pDNA produced per biomass. This illustrates the relevance of the flux to the PPP for pDNA synthesis.

### 3.4. Flux Balance Analysis of pDNA Production in Strain VH36

**Figure 4:**
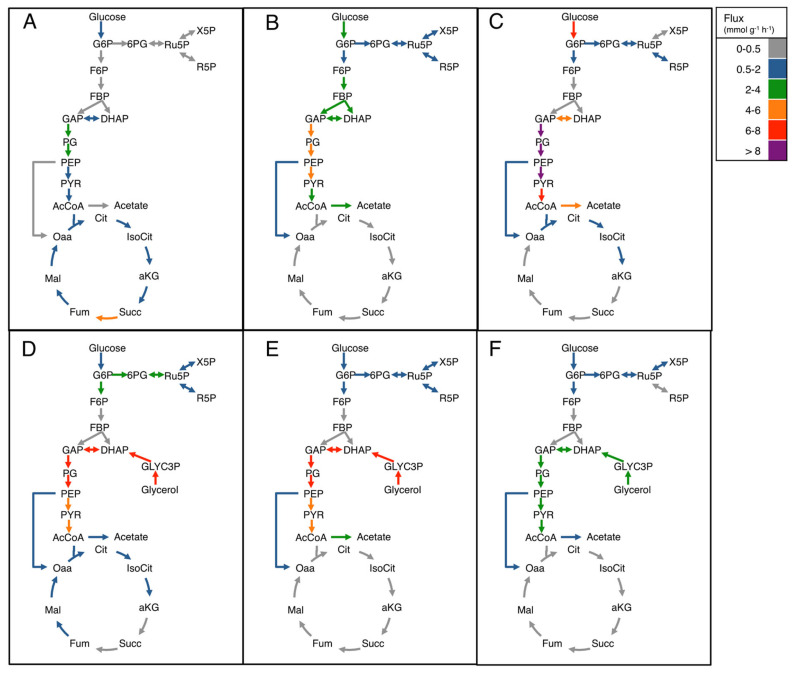
Estimated metabolic fluxes during different pDNA production strategies. (**A**): strain VH36 in glucose as the only C-source; (**B**): strain VH36rpiA in glucose as the only C-source; (**C**): strain VH36zwf in glucose as the only C-source; (**D**): strain VH36 co-consuming glucose and glycerol; (**E**): strain VH36rpiA co-consuming glucose and glycerol; (**F**): strain VH36zwf co-consuming glucose and glycerol.

To better understand the physiological changes caused by the different strategies used to improve pDNA production, metabolic flux analysis was performed. Figure 4 shows the estimated fluxes under the different conditions. When glucose was the only C-source, overexpressing genes from the PPP caused an increase in the *q_glucose_* (Figure 4B,C, compared to Figure 4A). The fluxes in the first reaction of the PPP, catalyzed by the G6PDH, were estimated to increase from 0.35 mmol g^−1^ h^−1^ in VH36, to 1.51 and 0.90 mmol g^−1^ h^−1^ in strains VH36rpiA and VH36zwf, respectively. The flux from D-ribulose 5-phosphate (Ru5P) to D-ribose 5-phosphate (R5P), catalyzed by RpiA, also increased due to *zwf* or *rpiA* overexpression using glucose exclusively. Namely, the estimated flux was 0.20 mmol g^−1^ h^−1^ in VH36 and increased to 0.70 and 0.64 mmol g^−1^ h^−1^ in VH36rpiA and VH36zwf, respectively. Therefore, the overexpression of *zwf* or *rpiA* in glucose as the C-source efficiently increased the metabolic fluxes through the PPP.

The simultaneous consumption of glucose and glycerol also increased the flux through the PPP. The flux to 6-phosphogluconolactone (6PG) from glucose 6-phosphate (G6P), catalyzed by the G6PDH, was 2.55 mmol g^−1^ h^−1^ in strain VH36 (Figure 4D), which is seven-fold greater than in cultures with glucose as the only C-source and the highest value estimated for all the different conditions. Overexpression of *rpiA* or *zwf* in a mixture of glucose and glycerol increased the above-mentioned flux only by 10 and 20%, respectively (Figure 4E,F), in comparison with cultures overexpressing those genes using glucose as the only C-source. The model also estimates a six-fold increase in the flux through the RpiA when strainVH36 is cultured in the C-source mixture, while it only increased by 30% when the *rpiA* gene was overexpressed, and decreased 20% when *zwf* was overexpressed, compared to the same genes overexpressed using glucose as the only C-source. It was estimated that the fluxes through the TCA cycle decreased compared to cultures of VH36 in glucose (Figure 4A), in cultures of VH36rpiA in glucose and glucose-glycerol mixtures (Figure 4B and Figure 4E, respectively), and in cultures of VH36zwf in the glucose-glycerol mixture (Figure 4F). In the cases of VH36zwf in glucose (Figure 4C) and VH36 in glucose-glycerol mixtures (Figure 4D), it seems that the TCA cycle tends to accumulate alpha-ketoglutarate (aKG). Interestingly, these two cases correspond to the best conditions for pDNA production.

## 4. Discussion

The demand for high-quality pDNA is expected to keep increasing due to the advances in gene therapy, mRNA, and DNA vaccines. Therefore, strategies leading to the intensification of bioprocesses for pDNA production are needed. pDNA production faces the typical challenges of *E. coli* fermentations, like overflow metabolism. Reconfiguring the glucose uptake in *E. coli* can drastically reduce the overflow metabolism, and high cell-densities can be attained in batch mode [27]. Particularly, the substitution of the phosphotransferase system by the galactose permease in the strain VH33 resulted beneficially for pDNA production in high cell-density cultures in batch mode [12]. Further cell engineering [13] and culture medium optimization [28] increased the pDNA yields of strain VH33. The additional deletion of the gene *pykA* in strain VH34 increased the pDNA yields in chemostat studies [15]. Due to the low overflow metabolism and the possibility of attaining high cell-densities in batch mode or increasing the pDNA yield at low dilution rates, this study focused on improving the production of pDNA in strain VH34.

The replication of a plasmid imposes a metabolic burden in the host, characterized by a growth rate decrease and downregulation of the expression of genes related to biosynthesis, energy generation, and glycolytic pathways [29]. It has been reported that the *zwf* gene was slightly overexpressed as a response to the metabolic burden caused by plasmid maintenance [29]. The PPP is one of the most important metabolic pathways because it contributes to maintaining the proper functioning of the cell, and supplies precursors for the synthesis of amino acids and nucleotides. The PPP is also an important source of NADPH, which is necessary for the cell to fight oxidative stress and to fuel the synthesis of many other molecules [30]. Increasing the flux in the PPP has improved the production of biomolecules in several microorganisms [31,32]. Because the PPP generates intermediates for the synthesis of nucleotides, as well as NADPH, several researchers have proposed that increasing the flux in this pathway can be useful to ameliorate the metabolic burden imposed by plasmid replication. For instance, Flores and coworkers [9] reported that overexpression of the *zwf* gene resulted in a growth rate increase of plasmid-bearing cells. However, the authors did not quantify the pDNA produced. The characteristics of strain VH34 make it a potential host for pDNA production processes. Due to the engineered glucose transport system, overflow metabolism is efficiently prevented (Table 1). A lower flux through the pyruvate kinase resulting from the *pykA* deletion is desirable to increase pDNA yields [33,34], which was confirmed here (Table 2). Yet, the growth rate of strain VH34 is low compared to its parental strain, and the quality of the pDNA produced is poor. The latter factor was improved by deleting the *recA* gene (strain VH36) but the SCF was still low, and the Y_pDNA/X_ decreased. It has been shown that the RecA protein has no effect on the supercoiling activity of the DNA gyrase GyrA. In contrast, RecA stimulates the relaxation activity of topoisomerase I [35]. Thus, the increased SCF in the *recA* mutants is an effect of decreased pDNA relaxation. The reduction of pDNA yields as an effect of *recA* inactivation has been observed for other strains [16,26]. The central role of *recA* in several physiological activities, like homologous recombination [36] and SOS response [37], has been described. However, the exact reasons for the decrease of Y_pDNA/X_ in *recA* mutants remain unclear. Another characteristic of strain VH34 (and its derivative VH36) is the capacity to consume glucose and an additional C-source simultaneously. Glycerol was provided with the aim of fueling the lower glycolysis, while glucose would be used in the upper glycolysis and possibly increasing the flux to the PPP. This effect was confirmed by a higher G6DPH activity and estimated flux, compared to the growth rate in glucose as the only C-source. The effects of combined C-sources were clear: although the growth and pDNA production was better in glycerol than in glucose, the simultaneous consumption of both C-sources resulted in additional improvements (Figure 2A). Moreover, the SCF increased importantly. The DNA gyrase is a topoisomerase that introduces negative DNA supercoiling in a process that requires ATP [38,39]. The simultaneous consumption of glycerol and glucose may increase the ATP generation rate compared to the cultures in glucose as the only C-source, which in turn could increase the gyrase activity.

The overexpression of *rpiA* in glucose did not increase Y_pDNA/X_, which contrasts with a previous report [10]; however, it increased the growth rate and SCF, which suggests that *rpiA* overexpression is also useful to reduce the metabolic burden and increase the energy generation rate in strain VH36. Consistent with previous reports [9], overexpressing *zwf* was an efficient way to increase the growth rate in glucose. Moreover, our results show that *zwf* overexpression also improved pDNA production in glucose, while Williams and coworkers reported no improvement [11]. Nevertheless, it should be noted that Williams and coworkers used a different strain, a purpose-defined medium, a fed-batch scheme, and thermal induction. Overexpression of *rpiA* or *zwf* in mixtures of glucose and glycerol drastically reduced the SCF. Although VH36 is a *recA*^−^ mutant, which is supposed to stabilize the supercoiled DNA, there are other mechanisms that can promote plasmid multimerization in *E. coli* [40]. Plasmid stability is affected by environmental factors like pH and dissolved oxygen [41,42]. It is possible that the overexpression of the gene and plasmid replication using the two C-sources triggers a stress condition that affects plasmid stability, which can lead to its multimerization [43]. A possible linked effect is the inhibition of site-specific recombinases, which are the enzymes that resolve multimeric plasmids [44]. However, the reasons for the plasmid multimerization only when mixed C-sources were used, and *zwf* or *rpiA* were overexpressed, remain unclear. The plasmid multimerization causes a reduction in the expression of the plasmid-encoded genes [45,46], which may explain the lower in vitro G6PDH activity measured for cultures overexpressing *zwf* in C-source mixtures (Figure 3A). The metabolic fluxes estimated by flux balance analysis show that the different strategies increased the flux to the PPP. The highest fluxes through the G6PDH were estimated in the conditions of higher pDNA production (VH36 grown in mixed C-sources, and overexpressing *zwf* in glucose). In these two conditions, the aKG formation rate is higher than its consumption rate in the TCA cycle (Figure 4C,D). This aKG surplus may be used for the synthesis of glutamine, which is required for the synthesis of deoxyadenosine triphosphate, deoxyguanosine triphosphate [1], thus contributing to the higher pDNA production observed.

In this study, it is demonstrated that the simultaneous consumption of glucose and glycerol is an easy and efficient strategy to increase the flux to the PPP and enhance pDNA production in strain VH36. Overexpression of the gene *zwf* in glucose can yield superior results. However, its implementation will require chromosomal expression to allow the production of any pDNA vector intended for gene therapy or DNA vaccination. The expression levels of *zwf* should be fine-tuned to avoid negative effects like the multimerization observed. This can be done by carefully selecting a combination of promoters and ribosome binding sites. Furthermore, it is desirable to achieve G6PDH activity levels higher than those reported in Figure 3B and prove whether a proportional increase of Y_pDNA/X_ can still be obtained. Moreover, it is desirable to experimentally determine whether there is a relationship between Y_pDNA/X_ and NADPH availability. Taken together, the results shown here are potentially useful for industrial pDNA production and product development.

## Figures and Tables

**Table 1:** Growth parameters of the untransformed strains W3110 and VH34 cultured in glucose, glycerol, or a mixture of glucose + glycerol. *µ*: specific growth rate; *q_glucose_*: specific glucose uptake rate; *q_glycerol_*: specific glycerol uptake rate; *q_acetate_*: specific acetate production rate; RQ: respiratory quotient.

C-Source	Strain	*µ* [h^−1^]	*q_glucose_* [g g^−1^ h^−1^]	*q_glycerol_* [g g^−1^ h^−1^]	*q_acetate_* [g g^−1^ h^−1^]	RQ [mol mol^−1^]
Glucose	W3110	0.53 ± 0.02	1.35 ± 0.44	-	0.17 ± 0.04	0.92 ± 0.05
	VH34	0.31 ± 0.01	0.55 ± 0.17	-	0.00 ± 0.00	1.11 ± 0.03
Glycerol	W3110	0.43 ± 0.02	-	2.06 ± 0.23	0.07 ± 0.00	0.79 ± 0.01
	VH34	0.27 ± 0.02	-	0.78 ± 0.12	0.00 ± 0.00	0.76 ± 0.12
Glucose + glycerol	W3110	0.46 ± 0.01	1.35 ± 0.17	-	0.26 ± 0.02	1.11 ± 0.02
	VH34	0.35 ± 0.01	0.48 ± 0.11	0.21 ± 0.08	0.04 ± 0.02	0.92 ± 0.06

**Table 2:** Main parameters of pDNA production of the strains W3110recA^−^, VH34, and VH36 during exponential growth in cultures in a mineral medium with glucose as the only C-source.

Strain	*µ* [h^−1^]	*q_glucose_* [g g^−1^ h^−1^]	*q_acetate_* [g g^−1^ h^−1^]	Y_pDNA/X_ [mg g^−1^]	*q_pDNA_* [mg g^−1^ h^−1^]	SCF [%]
W3110recA^−^	0.59 ± 0.01	1.47 ± 0.12	0.30 ± 0.02	1.68 ± 0.08	0.99 ± 0.13	78 ± 6
VH34	0.17 ± 0.00	0.50 ± 0.05	0.00 ± 0.00	1.92 ± 0.01	0.32 ± 0.00	4 ± 1
VH36	0.17 ± 0.00	0.31 ± 0.03	0.01 ± 0.00	1.21 ± 0.04	0.20 ± 0.01	39 ± 4

## Data Availability

Data are contained within the article.
